# Prognostic Relevance of Speckle Tracking-Derived Biatrial Stiffness Index in Patients with Dilated Cardiomyopathy

**DOI:** 10.3390/jcm15166167

**Published:** 2026-08-08

**Authors:** Aura Vîjîiac, Ioana Petre, Sebastian Onciul, Alina Scărlătescu, Diana Zamfir, Radu Gabriel Vătășescu

**Affiliations:** 1 Cardiology Department, “Carol Davila” University of Medicine and Pharmacy, 050474 Bucharest, Romania; aura.apostolescu@yahoo.com (A.V.); i_comanescu@yahoo.com (I.P.); 2 Emergency Clinical Hospital, 014491 Bucharest, Romania; alina.scarlatescu@gmail.com

**Keywords:** dilated cardiomyopathy, atrial stiffness, speckle tracking, atrial strain, biatrial stiffness index, prognosis

## Abstract

**Background:** Atrial stiffness can be estimated non-invasively using speckle-tracking echocardiography (STE) and has recently emerged as an outcome predictor. We aimed to assess left atrial (LA) and right atrial (RA) phasic function; the LA stiffness index (LASI), the RA stiffness index (RASI) and their sum; and the biatrial stiffness index (BASI) in dilated cardiomyopathy (DCM), and to test whether combining the two atria adds prognostic information over either index alone. **Methods:** A total of 121 patients with non-ischaemic DCM in sinus rhythm were followed prospectively for a composite endpoint of all-cause death, non-fatal cardiac arrest, or hospitalisation for heart failure decompensation. LASI was defined as the mitral E/e′ ratio divided by LA reservoir strain, RASI as the tricuspid Et/e′t ratio divided by RA reservoir strain, and BASI as the sum of the two. Cox models were adjusted for NYHA class, LV ejection fraction (LVEF), maximal LA volume (LAVmax) and pulmonary artery systolic pressure (PASP). **Results:** After 19 ± 11 months, 55 patients reached the endpoint. LA reservoir and contraction strain, all three components of RA strain and all three stiffness indices were significantly impaired in patients with events. All stiffness indices were independent outcome predictors in multivariable Cox regression (HR 2.79 [95% CI, 1.35–5.75], *p* = 0.006 for LASI, HR 1.84 [95% CI, 1.02–3.29], *p* = 0.04 for RASI and HR 2.74 [95% CI, 1.36–5.51], *p* = 0.005 for BASI). BASI showed the greatest increase in risk prediction (Δ likelihood ratio χ^2^ test = 10.3, *p* = 0.001) over NYHA class, left ventricular ejection fraction, LA maximal volume and pulmonary artery systolic pressure. BASI showed the highest discrimination (AUC = 0.73); however, it was not significantly better than LASI or RASI alone. **Conclusions:** Left and right atrial stiffness are both associated with adverse outcome in DCM and add prognostic information to an LV-centred risk model. Their unweighted sum performs at least as well as either component and offers a single parsimonious measure, whose incremental clinical value remains to be established in larger, externally validated cohorts.

## 1. Introduction

Dilated cardiomyopathy (DCM) is defined by left ventricular (LV) dilatation and global systolic dysfunction that cannot be explained by abnormal loading conditions or significant coronary artery disease [1,2]. It is one of the commonest causes of heart failure (HF) in young adults and remains the leading indication for heart transplantation. Prevalence estimates have risen substantially as ascertainment has improved, from the classical figure of approximately 1 in 2500 to as high as 1 in 250 in contemporary series. A genetic cause is identifiable in roughly a third of patients [3,4]. Despite the marked prognostic gains achieved with modern guideline-directed medical therapy [5], a substantial proportion of patients still progress to advanced HF, and LVEF alone discriminates outcome only modestly. Improved risk stratification therefore remains a clinically relevant goal.

Although DCM is regarded as a primary disorder of the LV myocardium, it produces remodelling and functional change in the right ventricle (RV) and in both atria, either as a haemodynamic consequence of LV failure or as part of the same myopathic process—consistent with the concept of an accompanying atrial cardiomyopathy [6]. There is a coherent mechanistic rationale for expecting atrial stiffness in particular to have prognostic value. Atrial stiffness represents the change in atrial pressure per unit change in atrial volume during passive atrial filling (the reservoir phase), in which the atrium fills from the pulmonary veins with the atrioventricular valve closed during ventricular systole [7]. Thus, it captures the reservoir properties of the atrium rather than its size alone. A stiffened LA cannot buffer rising filling pressures, which are transmitted to the pulmonary venous bed. Sustained elevation leads to pulmonary hypertension and increased RV afterload [8]. The resulting rise in RV filling pressures drives RA dilatation, hypocontractility and stiffening, establishing a left-to-right sequence in which both atria are ultimately affected. Because stiffness indices incorporate both filling pressure and deformation, they may detect impairment that precedes overt atrial enlargement [9,10]. Because the process involves both atria, an index integrating left- and right-sided stiffness is physiologically appealing.

Both left and right atrial dysfunction have emerged as prognostic markers in HF with reduced ejection fraction (EF) [11,12]. A non-invasive approach has been proposed which uses myocardial velocities from tissue Doppler imaging (TDI) together with atrial deformation to estimate the LA stiffness index (LASI) [13]. Data on the non-invasive RA stiffness index (RASI) are far more limited: to our knowledge, it has been reported only in systemic sclerosis [14], and not in a HF or DCM population. Whether combining left- and right-sided stiffness into a single measure adds prognostic information has not been formally tested. We have previously presented a preliminary analysis of a combined biatrial stiffness index (BASI), defined identically to the index used here, in 60 patients drawn from the present cohort, in abstract form [15]; the current study is therefore a substantially larger, fully adjusted and internally validated evaluation of that concept rather than its first description.

We hypothesised that (i) LASI and RASI are each associated with adverse outcome in DCM independently of established LV-based predictors, and (ii) because DCM alters the mechanics of both atria, a combined biatrial index would carry at least as much prognostic information as either component alone. Our pre-specified aims were to compare LA and RA phasic function and stiffness between patients with and without events; to test LASI, RASI and BASI as independent outcome predictors; to quantify their incremental value over an established risk model; and to compare the stiffness indices with parameters of atrial phasic function.

## 2. Materials and Methods

### 2.1. Study Population

We screened 158 consecutive adult patients with DCM who were in sinus rhythm and were referred to our echocardiography laboratory between January 2019 and December 2021. DCM was diagnosed using the following criteria: a dilated LV [16] with LVEF < 40%, in the absence of significant coronary artery disease or significant preload/afterload changes [17]. Exclusion criteria were a poor acoustic window (n = 7), severe orthopnoea (n = 18), comorbidities with life expectancy < 1 year (n = 2), and cor pulmonale (n = 10), leaving 121 patients in the final study population. Patients with severe orthopnoea were excluded by pre-specified design: these patients could not reliably maintain the left lateral decubitus position and breath-hold required for adequate acquisition and reliable strain tracking. The study protocol complied with the Declaration of Helsinki and was approved by the ethics committee of our hospital. All patients gave written informed consent at enrolment. Recorded clinical data included medication, New York Heart Association (NYHA) class at enrolment and N-terminal pro-brain natriuretic peptide (NT-proBNP) levels where clinically available.

### 2.2. Echocardiography

Two-dimensional (2D) comprehensive echocardiographic acquisitions were performed according to current recommendations [18], using a Vivid E9 (GE Vingmed, Horten, Norway) ultrasound machine. Offline data analysis was performed with dedicated software (EchoPAC BT 12).

**Figure 1:**
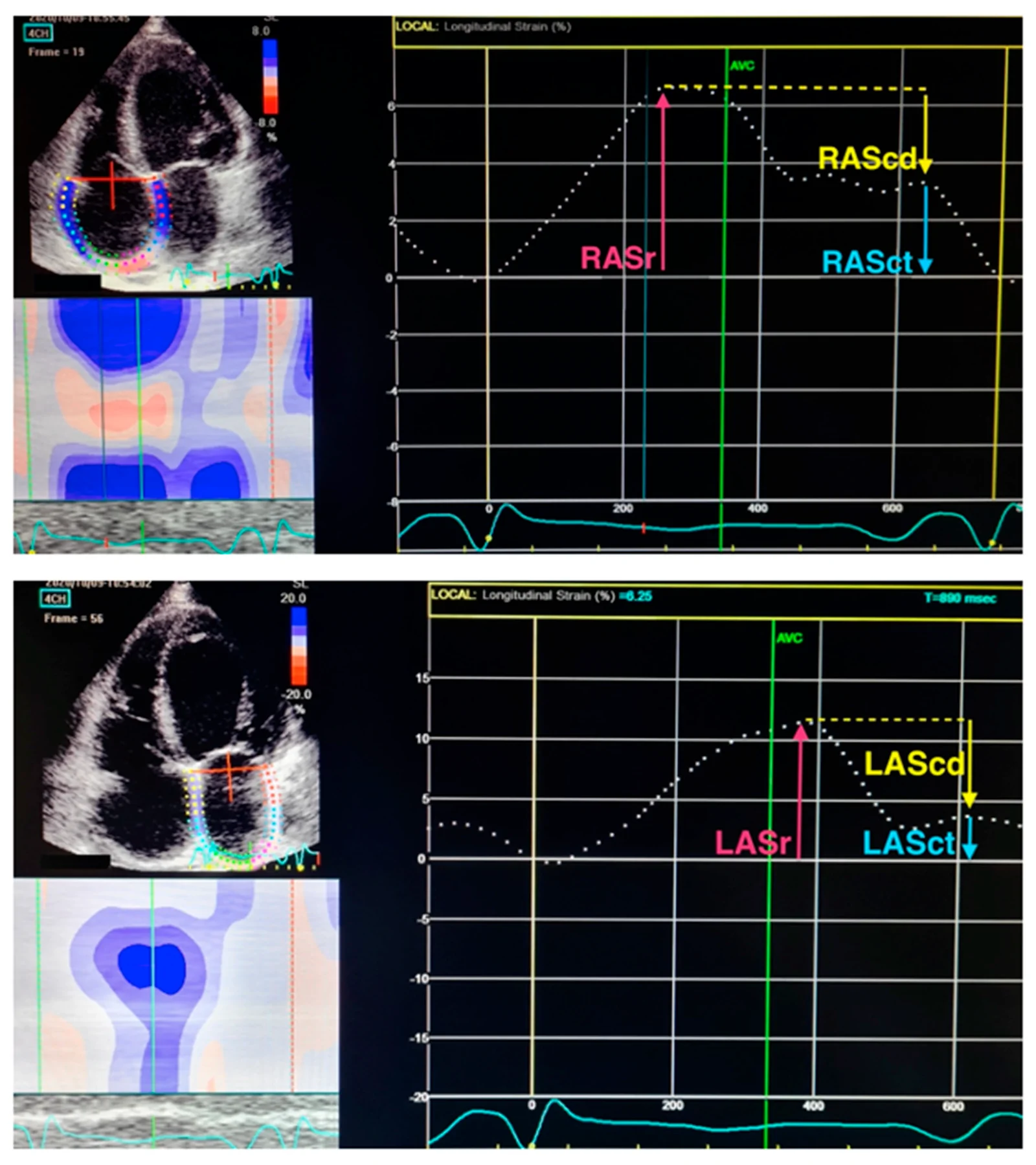
Apical four-chamber view with the average strain curve for the right atrium (**upper panel**) and left atrium (**lower panel**), used to measure reservoir strain (positive value), conduit and contraction strain (negative values). RASr—right atrial reservoir strain; RAScd—right atrial conduit strain; RASct—right atrial contraction strain; LASr—left atrial reservoir strain; LAScd—left atrial conduit strain; LASct—left atrial contraction strain.

The maximal LA and RA volumes (LAVmax and RAVmax) were obtained from the apical four-chamber view using the area–length method at ventricular end-systole. For atrial strain we used the apical four-chamber view and manually traced the endocardial border of the LA and RA, setting the zero-strain reference point at end-diastole, as recommended [19]. From the average strain curve of the atrial segments, we measured the three strain components of both atria (Figure 1), each reflecting one atrial phasic function [19]:

- The reservoir LA (LASr) and RA strain (RASr), reported as a positive value;
- The conduit LA (LAScd) and RA strain (RAScd), reported as a negative value;
- The contraction LA (LASct) and RA strain (RASct), reported as a negative value.

The intra- and interobserver reproducibility of atrial strain in our echocardiography laboratory has been published elsewhere [12].

### 2.3. Atrial Stiffness Indices

Using myocardial velocities and atrial strain derived from STE, we calculated three non-invasive indices of atrial stiffness:

- LASI, defined as the mitral E/e′ ratio divided by LASr, as previously reported [20];
- RASI, defined as the tricuspid Et/e′t ratio divided by RASr, as previously reported [14];
- BASI, defined as the arithmetic sum of LASI and RASI [15].

Since a higher E/e′ ratio reflects greater filling pressures and more severe diastolic dysfunction, while a lower atrial reservoir strain reflects more impaired atrial function, a higher LASI, RASI or BASI indicates more pronounced atrial stiffness.

### 2.4. Follow-Up and Endpoint

Patients were followed prospectively for 19 ± 11 months through office visits or telephone contact. The endpoint was a composite of all-cause death, non-fatal cardiac arrest, or hospitalisation for heart failure decompensation, whichever occurred first. Pre-specified definitions were as follows: all-cause death from any cause, ascertained from hospital records, or contact with next of kin; non-fatal cardiac arrest as documented sustained ventricular tachycardia or ventricular fibrillation requiring cardioversion, defibrillation or appropriate device therapy; and hospitalisation for heart failure decompensation as an unplanned overnight admission with worsening symptoms and signs of congestion requiring intravenous diuretic or vasoactive therapy. Outcomes were ascertained by a single investigator from medical records and telephone contact. Only the first qualifying event contributed to the composite endpoint, and time to event was measured from the baseline echocardiogram.

### 2.5. Statistical Analysis

Statistical analysis was performed using SPSS version 20.0 and GraphPad Prism version 10.0.0. We evaluated the normality of distribution using the Kolmogorov–Smirnov test. Continuous normal data are reported as mean (standard deviation) and compared with Student’s *t*-test; continuous non-normal data as median (interquartile range) and compared with the Mann–Whitney U test; categorical data as number (percentage) and compared with the χ^2^ test.

Receiver operating characteristic (ROC) curves and the respective area under the curve (AUC) were used to compare the ability of various parameters to predict adverse outcome. The Youden index was used to identify the optimal cutoff values for each variable.

Subjects who did not experience the event by the end of the follow-up period were treated as right-censored. Because many atrial indices were tested against the same endpoint, *p* values were corrected for multiple comparisons using the Benjamini–Hochberg false-discovery-rate procedure [21], and both raw *p* values and adjusted q values are reported. To control for confounding variables, a Cox proportional hazards regression analysis was used to calculate adjusted hazard ratios (HRs) with 95% confidence intervals (CIs). The proportional hazards assumption was tested using scaled Schoenfeld residuals. The pre-specified multivariable model included one atrial index at a time together with variables that are well-established predictors in DCM and had *p* < 0.10 in our univariable analysis. To examine the incremental prognostic value of the stiffness indices, we used the likelihood-ratio χ^2^ test for nested models and Harrell’s C-statistic. Statistical significance was defined as a two-tailed *p* value < 0.05.

## 3. Results

### 3.1. Clinical and Echocardiographic Characteristics

The general characteristics of the study population are presented in Table 1. All echocardiographic and clinical variables were complete for all 121 patients. NT-proBNP was available in only 57 out of 121 patients (47%) because it was measured on clinical indication rather than by protocol. During follow-up, 55 patients reached a major adverse cardiovascular event (MACE): there were 26 deaths (21%), 5 non-fatal cardiac arrests (4%), and 24 readmissions for heart failure decompensation (20%). Patients with events had more severe symptoms (*p* < 0.001 for NYHA class), higher NT-proBNP levels among those in whom it was measured (*p* = 0.008) and higher diuretic use (*p* = 0.002), consistent with an endpoint driven substantially by heart failure exacerbations.

The echocardiographic findings are presented in Table 2. Patients with events had more impaired LA reservoir and booster pump function, while all three components of RA strain were significantly more altered in patients with adverse outcome. Eighty-seven patients (72%) had mild mitral regurgitation (MR), 26 (21%) had moderate MR and 8 (7%) had severe MR, with no significant difference in MR severity between patients with and without events (*p* = 0.06). All three stiffness indices were significantly higher in patients with MACE, reflecting greater atrial stiffness. Tricuspid annular plane systolic excursion (TAPSE) and tricuspid annular systolic velocity (S′ wave) were also markedly lower in patients with events (both *p* < 0.001).

### 3.2. Prognostic Role of Atrial Phasic Function and Stiffness

We used univariable Cox regression to assess the prognostic power of various parameters (Table 3). All atrial volumes and strain indices, as well as LASI, RASI and BASI, were significant predictors of the composite endpoint, and all remained significant after false-discovery-rate correction.

Atrial parameters were introduced in ROC analysis to compare their predictive ability. Variables with satisfactory AUC (>0.60) are summarized in Table 4. BASI had the best AUC (AUC = 0.73, *p* < 0.001), followed by LASI (AUC = 0.71, *p* < 0.001) and RASI (AUC = 0.70, *p* < 0.001 for all). A value of BASI > 1.70 had 78% sensitivity and 72% specificity for event prediction. However, the CI of all the atrial indices overlapped substantially, and formal DeLong testing showed that BASI was not significantly better than LASI (*p* = 0.09) or RASI (*p* = 0.49).

Atrial strain and stiffness indices were then tested in multivariable Cox regression. We constructed a multivariable model which included one atrial index at a time, together with parameters which are well-established predictors in DCM and which had significant power in our univariable analysis (*p* < 0.10): NYHA class, LVEF, LAV_max_, pulmonary artery systolic pressure (PASP). BNP levels were not included in the analysis since they were not available for all patients. LASI, RASI and BASI were tested both as continuous and as dichotomous variables, using cut-offs derived from the Youden index. RASr, RAScd and all atrial stiffness parameters were independent predictors of adverse outcome, but the models including LASI and BASI yielded higher C-statistic (Table 5). A LASI > 1.10 and a BASI > 1.70 were both associated with a ~3-fold risk of MACE. This pattern, where stiffness indices survived adjustment and multiplicity correction while most of the phasic strain parameters did not, is one robust finding of the study.

**Figure 2:**
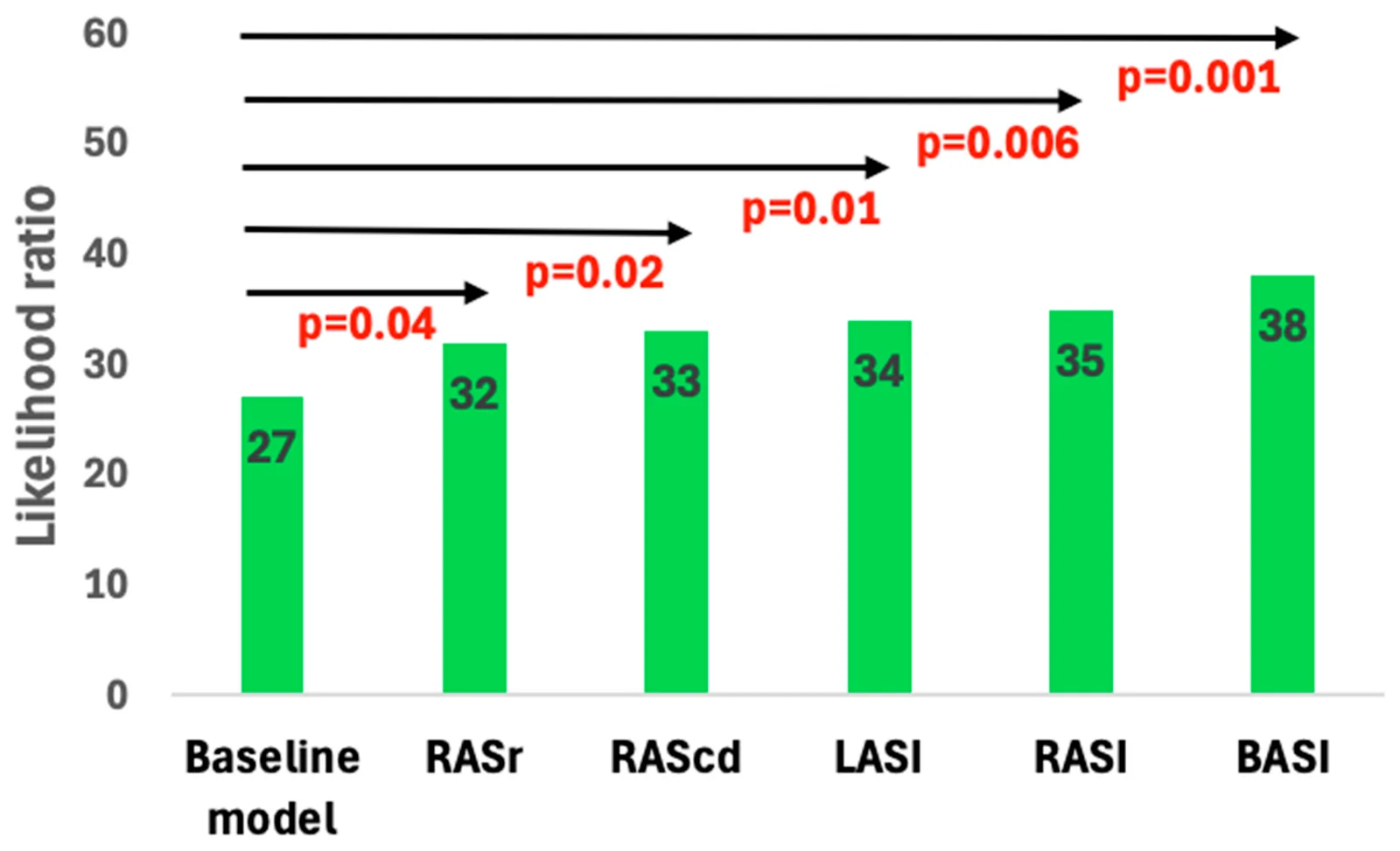
Bar charts showing the incremental prognostic value of atrial parameters over traditional prognostic factors presented as a likelihood ratio χ^2^ test. The addition of RASr, RAScd, RASI, LASI and BASI over the baseline model, which included NYHA class, LVEF, LA maximal volume and PASP, led to significant incremental prognostic power. Abbreviations are in text.

The likelihood-ratio χ^2^ test demonstrated incremental value over the baseline model for RASr (Δχ^2^ = 4.8, *p* = 0.04), RAScd (Δχ^2^ = 6.0, *p* = 0.02), LASI (Δχ^2^ = 6.3, *p* = 0.01), RASI (Δχ^2^ = 7.4, *p* = 0.006) and BASI (Δχ^2^ = 10.3, *p* = 0.001), with BASI yielding the largest increase (Figure 2). After false-discovery-rate correction across the nine indices, these increments remained significant for RAScd (q = 0.03), LASI (q = 0.03), RASI (q = 0.03) and BASI (q = 0.01) but not for RASr (q = 0.051).

RV systolic dysfunction is an established event predictor in DCM [22]. In our study, TAPSE was significantly more impaired in the subgroup that reached the endpoint (*p* < 0.001). TAPSE showed moderate correlation with LASI (r = −0.45, *p* < 0.001), RASI (r = −0.38, *p* < 0.001) and BASI (r = −0.51, *p* < 0.001), thus probably reflecting the pathophysiological link between RV systolic dysfunction and atrial stiffness. Given the relatively small number of events (55), we constructed a parsimonious multivariable model with only 5 variables (thus fulfilling the rule of 10), in order to reduce the risk of overfitting, while focusing on clinically relevant structural (LVEF, LA volume) and hemodynamic (PASP) confounders.

## 4. Discussion

In our study, DCM patients with adverse outcome had greater atrial dilation, more impaired LA reservoir and booster function, greater impairment of all three phasic functions of the RA and greater stiffness of both atria. Left and right atrial stiffness were both independent outcome predictors and, together with RASr and RAScd, were the only atrial parameters to survive both multivariable adjustment and correction for multiple testing. The combined BASI showed the highest point estimates of discrimination; however, it was not statistically superior to either of its components. We therefore present biatrial stiffness as a useful and parsimonious marker of risk in DCM, while avoiding any claim that the combined index is superior to its parts.

STE is a useful tool for the assessment of atrial phasic function and there are consistent prospective data reporting the prognostic role of LASr in heart failure with reduced EF [11,23,24]. We also found a predictive value for LASct on univariable analysis, which has been far less studied than LASr. Mandoli et al. recently found LASct to be an independent predictor of cardiovascular death and hospitalisation in outpatients with heart failure with reduced EF [25]. In our cohort, however, neither LASr nor LASct remained significant after multivariable adjustment, which tempers the emphasis we originally placed on phasic strain and is consistent with the view that deformation and filling pressure carry complementary rather than interchangeable information.

While RASr was found to correlate well with elevated RA pressure [26], data analysing RA phasic function in heart failure are scarce. Several cardiac magnetic resonance studies reported impairment of both RA reservoir and conduit function in patients with left heart failure [27,28]. In our study, all three components of RA phasic function were univariable outcome predictors in DCM, with RASr and RAScd retaining nominal significance and incremental value after adjustment. However, the utility of RA strain in clinical practice is still limited, partly because of the wide reference ranges for normal RA deformation parameters [29], and our findings warrant further research.

In the current study, the atrial parameters most robustly associated with MACE were the stiffness indices. Ventricular lusitropy does not depend only on atrial dimensions and contractility but also on atrial stiffness. From a pathophysiological standpoint, atrial stiffness represents the amount of pressure required to determine a unit change in atrial volume during ventricular diastole [7]. The non-invasive stiffness of the LA, using the mitral E/e′ ratio and LASr, has been scarcely studied over the last decade. Zhao et al. reported that increased LA stiffness was associated with subclinical organ damage in hypertensives, as LA stiffness precedes LA dilation and dysfunction [9]. Two recent studies found good correlation between non-invasive LASI and invasive LV end-diastolic pressure [20,30]. Kim et al. analysed 307 patients with heart failure and preserved EF retrospectively and found that non-invasive LASI was the best predictor of a composite of death or hospitalisation, with superior prognostic value to other parameters of diastolic function [31]. It is worth mentioning that the cut-off value for LASI in our study, 1.10, was higher than the optimal cut-off reported by Kim et al. of 0.26. A probable explanation is that patients with DCM and LV systolic dysfunction will most likely have greater LV filling pressures and probably greater LA dysfunction—and thus greater LA stiffness—than patients with preserved LVEF. In another recent study, Bytyçi et al. investigated 215 outpatients with LVEF < 50% and found that a LASI greater than 0.76 was the best predictor of death or hospitalisation [13]. The considerable dispersion of these published cut-offs reinforces the conclusion that no threshold for LASI can yet be recommended for clinical use.

A stiffened LA cannot accommodate increased intracavitary pressure and will lead to pulmonary venous congestion. In case of long-standing changes, this condition will determine irreversible pulmonary hypertension and increase the afterload of the RV [8]. Importantly, right ventriculo-arterial coupling itself has been shown to carry prognostic significance in heart failure [32]. RV dysfunction will raise intracavitary filling pressures and determine morpho-functional changes in the RA, which—in a similar manner to the LA—will become enlarged, hypokinetic and stiffened. The only previous study investigating RA stiffness included 70 patients with systemic sclerosis, in whom non-invasive RASI was the best predictor of all-cause death after a follow-up of 4.7 years [14].

The prognostic power of atrial stiffness might be explained by the formula used for its non-invasive assessment. It includes ventricular filling pressures expressed as the E/e′ ratio, which is itself an outcome predictor [33,34]. It also incorporates atrial reservoir strain, allowing LASI and RASI to reflect early changes in atrial mechanics which appear prior to atrial enlargement. This may at least partly explain why the stiffness indices had incremental prognostic power in our study while atrial volumes did not. Our findings support the view that biatrial mechanics have an independent pathophysiological role in left-sided HF, an idea that has been suggested previously [35].

The interpretation of our findings requires particular caution. Although BASI had the highest AUC and the largest likelihood-ratio increment, the model containing BASI was not significantly superior to the models containing LASI or RASI alone on formal DeLong testing. Since BASI is, by construction, the sum of its two components, it is not an independent variable, and comparisons against models already containing one component have limited power by design. The most defensible reading of our data is that much of the prognostic information in BASI is already present in each individual index, and that BASI is best regarded as a convenient summary of biatrial stiffness rather than as a superior predictor. Consistent with this, entering LASI and RASI as two separate covariates gave virtually the same discrimination as their sum. Moreover, the magnitude of the incremental gain is modest in absolute terms. We therefore regard biatrial stiffness as a promising risk marker whose clinical utility is not yet established, rather than as a tool ready for incorporation into risk scores.

We did not add parameters of RV function in our multivariable model to avoid overfitting (given the small number of events). Establishing whether atrial stiffness carries information independent of the RV will require larger cohorts able to support simultaneous adjustment, and ideally invasive validation.

Finally, we wish to be explicit about the provenance of the index. The definition of LASI, RASI and BASI used here, and the concept of summing left- and right-sided stiffness, were presented by our group in abstract form at the European Society of Cardiology Congress in 2022, in a preliminary analysis of 60 patients [15]. Those 60 patients are a subset of the 121 reported here, drawn from the same prospective registry and the same enrolment window. The cohorts therefore overlap completely rather than being independent. That earlier report used a narrower endpoint (heart failure rehospitalisation alone), a different adjustment set and no correction for multiplicity. It concluded that BASI provided better stratification than either component—a conclusion which the present, larger and more rigorously analysed dataset does not support. Therefore, we regard the present work as a consolidation and partial correction of that preliminary finding.

Much of the prognostic information of BASI may already be reflected by each individual atrial stiffness index. DCM is characterized by chronic elevation in left-sided filling pressures leading to LA stiffness, while RA remodelling may capture the downstream hemodynamic burden better than LA stiffness alone. Larger studies are required to establish whether BASI consistently provides incremental value over LASI and RASI, or whether a single atrial stiffness index is enough for proper risk stratification in DCM.

## 5. Study Limitations

As this was a single-centre study, its main limitation is the modest sample size, with a relatively short follow-up period. With 55 events the study supports approximately 11 events per variable in the primary models, which is adequate but leaves limited power for the more heavily adjusted models, and confidence intervals around all discrimination metrics are correspondingly wide. Second, and most importantly, all analyses were performed in a single cohort: cut-offs were derived and evaluated in the same patients, and no internal or external validation were possible. Therefore, the cut-offs we report should not be applied clinically until validated in an independent population. Third, there is a potential selection bias since we excluded patients with severe orthopnoea and patients with poor acoustic window. This means that our findings reflect ambulatory, compensated, technically imageable patients and they may not extend to acutely decompensated patients—in whom, in any case, the loading dependence of E/e′ and atrial strain would make stiffness estimation unreliable. Fourth, atrial stiffness was estimated non-invasively and was not validated against invasive pressure-volume measurements in this cohort.

## 6. Conclusions

Greater left and right atrial stiffness, estimated non-invasively by combining tissue Doppler velocities with atrial reservoir strain, was independently associated with major adverse cardiovascular events in patients with DCM. The unweighted biatrial sum, BASI, improved discrimination over a model containing NYHA class, LVEF, LA volume and PASP. It performed at least as well as either component; however it was not statistically superior to LASI or RASI alone. Biatrial stiffness is therefore a promising and easily obtained marker of risk in DCM whose incremental clinical value remains to be established in larger, externally validated cohorts.

## Figures and Tables

**Table 1:** Baseline clinical characteristics.

Variable	All Patients (n = 121)	MACE (n = 55)	No MACE (n = 66)	*p*
Age (years)	59 (14)	59 (15)	59 (14)	0.92
Male, n (%)	89 (74%)	40 (73%)	49 (74%)	0.85
NT-proBNP (pg/mL)	480 (163–1525)	690 (211–1815)	253 (105–505)	0.008
NYHA class, n (%)				<0.001
II	71 (59%)	22 (40%)	49 (74%)	
III	43 (35%)	27 (49%)	16 (24%)	
IV	7 (6%)	6 (11%)	1 (2%)	
Medication, n (%)				
ACE-I/ARB/ARNI	115 (95%)	52 (95%)	63 (95%)	0.82
β-blockers	117 (97%)	51 (93%)	66 (100%)	0.04
MRA	108 (89%)	51 (93%)	57 (86%)	0.26
SGLT2 inhibitors	17 (14%)	7 (13%)	10 (15%)	0.70
Loop diuretics	79 (65%)	44 (80%)	35 (53%)	0.002

Abbreviations are found in the text.

**Table 2:** Two-dimensional echocardiographic characteristics.

Variable	All Patients (n = 121)	MACE (n = 55)	No MACE (n = 66)	*p*
LVEDV (mL)	243 (96)	256 (104)	232 (89)	0.19
LVESV (mL)	185 (84)	198 (91)	174 (77)	0.13
LVEF (%)	25 (7)	24 (7)	26 (7)	0.08
LA Vmax index (mL/m^2^)	52 (25)	61 (25)	45 (22)	<0.001
LASr (%)	11.0 (6.4–16.5)	9.1 (5.0–12.3)	13.1 (8.1–20.7)	0.002
LAScd (%)	−5.1 (−8.6 to −3.1)	−4.5 (−6.6 to −2.8)	−5.5 (−9.4 to −3.4)	0.07
LASct (%)	−5.1 (−10.0 to −2.4)	−3.3 (−7.2 to −1.8)	−7.6 (−11.9 to −3.7)	<0.001
TAPSE (mm)	17 (15–20)	16 (12–18)	19 (17–23)	<0.001
S′ wave (cm/s)	11 (3)	9 (2)	12 (2)	<0.001
RA Vmax index (mL/m^2^)	29 (20–40)	34 (21–46)	25 (18–37)	0.02
RASr (%)	17.0 (10.8)	13.0 (8.8)	20.2 (11.2)	<0.001
RAScd (%)	−7.5 (6.0)	−5.6 (4.3)	−9.1 (6.7)	<0.001
RASct (%)	−9.5 (7.1)	−7.5 (6.3)	−11.1 (7.4)	0.004
PASP (mm Hg)	41 (17)	45 (18)	38 (16)	0.04
LASI	1.40 (0.61–2.78)	2.00 (1.18–3.81)	1.01 (0.56–1.85)	<0.001
RASI	0.38 (0.23–0.77)	0.49 (0.32–1.24)	0.31 (0.21–0.47)	<0.001
BASI	2.00 (0.99–3.52)	3.10 (1.76–5.21)	1.23 (0.82–2.53)	<0.001

Normally distributed variables are given as mean (standard deviation) and compared with Student’s *t*-test; non-normally distributed variables as median (interquartile range) and compared with the Mann–Whitney U test. Abbreviations are found in the text.

**Table 3:** Univariable Cox regression for the composite endpoint.

Variable	HR (95% CI)	*p*	q (BH)
NYHA class	2.381 (1.600–3.543)	<0.001	–
LVEF	0.951 (0.914–0.990)	0.014	–
LAVmax index	1.015 (1.006–1.023)	<0.001	–
LASr	0.936 (0.899–0.975)	0.002	0.002
LAScd	1.083 (1.005–1.167)	0.035	0.035
LASct	1.113 (1.045–1.186)	<0.001	0.001
RAVmax index	1.013 (1.004–1.021)	0.004	–
RASr	0.949 (0.921–0.978)	<0.001	0.001
RAScd	1.108 (1.042–1.177)	<0.001	0.001
RASct	1.063 (1.013–1.115)	0.014	0.015
PASP	1.013 (0.999–1.028)	0.067	–
TAPSE	0.806 (0.749–0.867)	<0.001	–
S′ wave	0.694 (0.617–0.779)	<0.001	–
LASI	1.300 (1.168–1.447)	<0.001	0.000
RASI	1.359 (1.170–1.579)	<0.001	0.000
BASI	1.261 (1.160–1.371)	<0.001	0.000

HR—hazard ratio; CI—confidence interval; q (BH)—Benjamini–Hochberg false-discovery-rate adjusted *p* value, computed across the nine atrial indices only. The remaining abbreviations are found in the text.

**Table 4:** ROC analysis for the composite endpoint.

Variable	AUC (95% CI)	*p*
LAVmax index	0.70 (0.60–0.79)	<0.001
LASr	0.67 (0.57–0.77)	0.001
LASct	0.70 (0.60–0.79)	<0.001
RAVmax index	0.63 (0.53–0.73)	0.02
RASr	0.70 (0.61–0.79)	<0.001
RAScd	0.67 (0.58–0.77)	0.001
RASct	0.66 (0.56–0.76)	0.002
LASI	0.71 (0.61–0.80)	<0.001
RASI	0.70 (0.61–0.80)	<0.001
BASI	0.73 (0.64–0.82)	<0.001

**Table 5:** Multivariable Cox regression models for the composite endpoint.

Multivariable Model	HR (95% CI)	*p*	C-Statistic (95% CI)
Baseline model + LASr (%)	0.97 (0.92–1.01)	0.14	0.70 (0.62–0.77)
Baseline model + LAScd (%)	1.04 (0.96–1.13)	0.32	0.69 (0.62–0.77)
Baseline model + LASct (%)	1.05 (0.98–1.13)	0.16	0.70 (0.63–0.78)
Baseline model + RASr (%)	0.97 (0.94–1.00)	**0.048**	0.71 (0.63–0.78)
Baseline model + RAScd (%)	1.07 (1.01–1.14)	**0.03**	0.71 (0.64–0.78)
Baseline model + RASct (%)	1.03 (0.98–1.08)	0.29	0.70 (0.63–0.77)
Baseline model + LASI	1.19 (1.05–1.35)	**0.008**	0.72 (0.65–0.79)
Baseline model + LASI, cutoff > 1.10	2.79 (1.35–5.75)	**0.006**	0.72 (0.65–0.79)
Baseline model + RASI	1.35 (1.12–1.61)	**0.001**	0.71 (0.64–0.78)
Baseline model + RASI, cutoff > 0.76	1.84 (1.02–3.29)	**0.04**	0.70 (0.62–0.77)
Baseline model + BASI	1.19 (1.08–1.31)	**0.001**	0.72 (0.66–0.79)
Baseline model + BASI, cutoff > 1.70	2.74 (1.36–5.51)	**0.005**	0.71 (0.64–0.78)

Baseline model includes NYHA class, LVEF, LAVmax and PASP. Bolded *p* values are statistically significant, HR—hazard ratio; CI—confidence interval. The rest of the abbreviations are found in text.

## Data Availability

The de-identified dataset and the analysis code supporting the findings of this study are available from the corresponding author on reasonable request.
